# Hyperhomocysteinemia predicts renal function decline: a prospective study in hypertensive adults

**DOI:** 10.1038/srep16268

**Published:** 2015-11-10

**Authors:** Di Xie, Yan Yuan, Jiangnan Guo, Shenglin Yang, Xin Xu, Qin Wang, Youbao Li, Xianhui Qin, Genfu Tang, Yong Huo, Guangpu Deng, Shengjie Wu, Binyan Wang, Qin Zhang, Xiaobin Wang, Pu Fang, Hong Wang, Xiping Xu, Fanfan Hou

**Affiliations:** 1 National Clinical Research Center of Kidney Disease, State Key Laboratory of Organ Failure Research, Division of Nephrology, Nanfang Hospital, Southern Medical University, Guangzhou, China; 2 School of Health Administration, Anhui Medical University, Hefei, China; 3 Department of Cardiology, Peking University First Hospital, Beijing, China; 4 Institute of Biomedicine, Anhui Medical University, Hefei, China; 5 Department of Population, Family and Reproductive Health, Johns Hopkins University Bloomberg School of Public Health, Baltimore, USA; 6 Department of Pharmacology, Center for Metabolic Disease Research, Temple University School of Medicine, Philadelphia, Pennsylvania, USA

## Abstract

Hyper-homocysteinemia (HHcy) is associated with microalbuminuria and glomerular injury in general and diabetic populations. However, HHcy’s role in hypertensive patients was not studied. We investigated whether HHcy is an independent risk factor for renal function decline and development of chronic kidney disease (CKD) in hypertensive men and women. This was a community-based prospective cohort study of 2,387 hypertensive adults without CKD at baseline, with a mean follow-up of 4.4 years. Baseline and follow-up levels of plasma Hcy, folate, vitamin B12, blood pressure and other pertinent covariables were obtained. CKD was defined as an estimated glomerular filtration rate (eGFR) <60 ml/min/per 1.73 m^2^ and an eGFR decline rate >1 ml/min/per 1.73 m^2^/year. There was a graded association between Hcy tertiles and eGFR decline. Subjects in the 3^rd^ tertile of Hcy levels had an accelerated rate of eGFR decline and an increased risk of incident CKD, as compared with those in the 1st tertile, after adjusting for age, gender, baseline diabetes, SBP, BMI, smoking, dyslipidemia, eGFR, folate and vitamin B12 levels. In conclusion, in this prospective cohort of Chinese hypertensive adults, elevated baseline plasma Hcy can serve as an independent biomarker to predict renal function decline and incident CKD.

Chronic kidney disease (CKD) is a serious clinical and public health challenge globally^1^,^2^,^3^. In China, 147 million people are affected by CKD^3^. Hypertension is also one of the leading causes of end stage renal disease (ESRD). Several studies have shown that even modestly elevated blood pressure (BP) places individuals at increased risk of ESRD relative to those with normal BP^4^,^5^,^6^. Therefore, early intervention to prevent renal injury in hypertensive population is of immense significance to prevent progression to CKD and ESRD. In addition to the traditional risk factors, such as age, BP, diabetes, smoking and hyperlipidemia, elevated plasma homocysteine (Hcy) levels, termed as hyperhomocysteinemia (HHcy), has emerged as an independent risk factor for the progression of CKD^7^,^8^. Experimental studies suggest that HHcy induces glomerular injury through redox signaling pathways and DNA hypomethylation mechanisms^9^,^10^,^11^,^12^,^13^. More importantly, recent human studies have shown that HHcy is associated with microalbuminuria in the general population and in patients with diabetes^8^,^14^,^15^. However, the effect of HHcy on renal function decline and incident CKD in hypertensive adults has not been studied.

Hcy is a sulfur-containing amino acid and an intermediate metabolite in methionine metabolism. Deficiency in Hcy metabolic enzymes and its co-factors, such as folate and other B vitamins, could lead to increased Hcy levels in both humans and mice^16^. It is known that methylenetetrahydrofolate reductase (MTHFR) is a key enzyme in folic acid metabolism that provides a methyl group for Hcy remethylation. The MTHFR 677TT variant impairs MTHFR function leading to the development of HHcy^17^. Because folate and other B vitamins are cofactors in the remethylation and trans-sulfuration pathways of Hcy metabolism, the supplementation of these vitamins has been used as an effective and inexpensive strategy for Hcy-lowering therapy^18^,^19^.

It is estimated that hypertensive disease affects 266 million adults in China, affecting nearly 27% of the adult population^20^. The Chinese population has a high prevalence of HHcy, particularly in the northern rural areas^21^,^22^, mostly due to a combination of low dietary folate intake and a higher prevalence of MTHFR 677TT mutation than in western populations (25% vs. <10%)^23^. Those with a high prevalence of folic acid deficiency (~40%) and hyperhomocysteinemia (~28%)^21^,^22^ might be at a high risk of vascular and renal disease as posed by HHcy and may benefit more from B vitamins supplementation. Recently, we demonstrated the efficacy of folic acid supplementation in primary prevention of stroke among adults with hypertension in China^24^. Therefore, studies to investigate the relationship between HHcy, B vitamins levels, renal function decline and incident CKD in hypertensive adults would help to identify potential risk factors or protectors in the prevention and treatment of CKD in this high-risk population, and are of great clinical and public health interest in both the Chinese population and populations elsewhere that share similar characteristics.

In this community-based prospective cohort study, we sought to investigate the independent effect of baseline Hcy, folate and vitamin B12 levels on renal function decline and incident CKD in Chinese hypertensive adults without baseline CKD.

## Results

### Cohort description

Data from 2,387 Chinese hypertensive adults with a mean age of 60 years (range: 45 to 76 years) and mean estimated glomerular filtration rate (eGFR) of 93.9 ml/min/per 1.73 m^2^ at baseline were analyzed. The mean blood pressure at baseline and at the end of the follow-up was 168 ± 98 and 152 ± 84 mmHg, respectively. Forty-four percent of participants received antihypertensive treatment. The percentage of participants achieving the target BP of <140/90 mmHg at the end of the follow-up was 21.2%. At baseline, men tended to be older, more likely to be a current smoker, and had higher DBP and lower serum total cholesterol, triglycerides and eGFR levels (Table 1). The median of plasma Hcy at baseline was 12.1 μmol/L, with a significant difference between men and women (P < 0.001). Gender differences were also found for folate levels. Meanwhile, no significant differences were found for baseline vitamin B12 levels between genders.

### Higher Hcy level is associated with increased risk for poor renal outcomes

**Figure 1:**
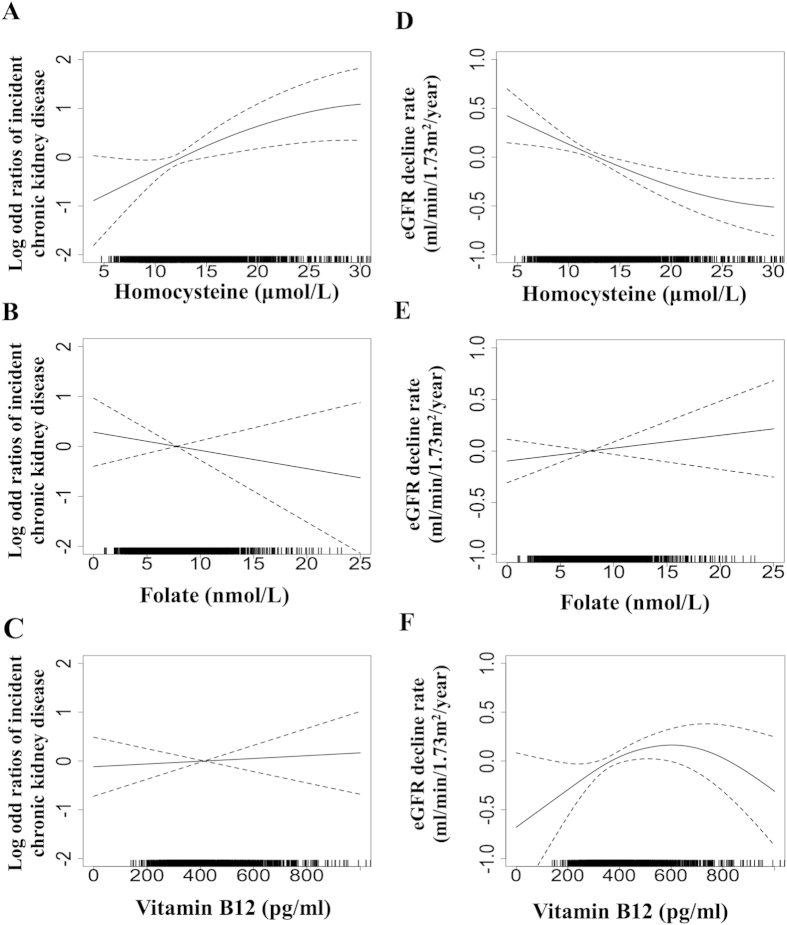
Adjusted splines of baseline homocysteine, folate and vitamin B12 and renal outcome. The solid line represents the spline of baseline homocysteine (**A**) folate (**B**) and vitamin B12 (**C**) and incident chronic kidney disease adjusted by age, gender, systolic blood pressure, diabetes, smoking, cholesterol, HDL-C, triglycerides, body mass index, estimated glomerular filtration rate (eGFR), and previous use of ACEIs/ARBs. Adjusted splines of baseline homocysteine (**D**), folate (**E**) vitamin B12 (**F**) and eGFR decline rate are also shown. Dotted lines represent the 95% confidence interval of the splines. Incident chronic kidney disease was defined as follow-up eGFR <60 ml/min/1.73 m^2^ and eGFR decline rate >1 ml/min/1.73 m^2^/year. eGFR decline rate was calculated as the difference between the follow-up eGFR and baseline eGFR, which was divided by follow-up duration and expressed as ml/min/per 1.73 m^2^/year. Negative eGFR change indicates a fall in eGFR during follow-up. Abbreviations: ACEIs, angiotensin converting enzyme inhibitors; ARBs, angiotensin II receptor blockers; eGFR, estimated glomerular filtration rate; HDL-C, high density lipoprotein cholesterol.

During 10,554 person-years (mean follow-up: 4.4 years), 69 (2.9%) participants developed CKD. As shown in Fig. 1A, a higher Hcy level at baseline was associated with higher incident CKD in the spline analysis. However, folate and vitamin B12 were not significantly associated with incident CKD in the spline analysis (Fig. 1B,C).

When analyzed as a continuous variable, higher log-transformed Hcy was associated with higher incident CKD (Table 2), even after adjusting for age, gender, diabetes, SBP, body mass index (BMI), smoking, cholesterol, high density lipoprotein cholesterol (HDL-C), triglycerides, eGFR, and previous use of angiotensin converting enzyme inhibitors (ACEIs)/angiotensin receptor blockers (ARBs). The association between Hcy levels and incident CKD remained unchanged after further adjustment for SBP at the end of the follow-up (Table 2). In analyses that considered tertiles of Hcy, the odds ratio (OR) for incident CKD increased across tertiles of plasma Hcy level, reaching 2.46 (95% confidence interval [CI] 1.05–5.77) for the top tertile compared with the lowest tertile (p for trend 0.011) in fully adjusted models.

Folate and vitamin B12 were not significantly associated with incident CKD in either unadjusted or adjusted models, respectively (Table 2). When Hcy, folate and vitamin B12 were all included in the multivariate model, only Hcy was significantly associated with an increased risk for incident CKD (Table 2).

### Higher Hcy level is associated with more rapid decline in renal function

The mean yearly change for eGFR in the cohort was −0.87 (Q1–Q3: −2.02 ~ 0.20) ml/min per 1.73 m^2^/year. As shown in Fig. 1D, a higher Hcy level at baseline was associated with faster rate of eGFR decline in the spline analysis. However, folate and vitamin B12 were not significantly associated with a faster rate of eGFR decline in the spline analysis (Fig. 1E,F).

Higher Hcy level was associated with a faster rate of eGFR decline in fully adjusted models (Table 3). The highest tertile of Hcy was also associated with a more rapid eGFR decline rate in multivariate models (Table 3). Folate was not significantly associated with a faster rate of eGFR decline in any of the models (Table 3). Higher levels of vitamin B12 were associated with a slower rate of eGFR decline (Table 3). However, when Hcy, folate and vitamin B12 were all included in the multivariate model, only Hcy was significantly associated with a faster rate of eGFR decline (Table 3).

### Stratified analysis

The association between higher log-transformed Hcy and increased risk of incident CKD appeared to be consistent across the subgroups, although the magnitude of association varied slightly. For example, the association between Hcy and CKD was stronger among those with baseline eGFR between 60–90 ml/min/per 1.73 m^2^ as compared to those with eGFR>90 ml/min/per 1.73 m^2^. However, tests for interaction were not significant (p for interaction >0.05) (Table 4). In addition, these associations were also found among subgroups stratified according to age, gender, baseline SBP and eGFR (p for interaction >0.05) (Table 4). In addition, a higher level of Hcy was associated with a more rapid eGFR decline rate among subgroups stratified according to age, gender, baseline SBP and eGFR (p for interaction >0.05) (Table 4). However, folate and vitamin B12 were not significantly associated with higher incident CKD or a faster rate of eGFR decline among any of the subgroups.

## Discussion

In this prospective study of 2,387 hypertensive adults, we identified a graded association between plasma Hcy levels and an increased risk of accelerated renal function decline and incident CKD. This association was independent of plasma folate and vitamin B12 levels and other traditional risk factors. Our findings suggest that elevated plasma Hcy levels, but not low folate and/or vitamin B12 levels, may predict progression of renal function decline and incident CKD in hypertensive adults.

Increased plasma levels of Hcy, termed hyperhomocysteinemia, is diagnosed when plasma Hcy levels are greater than 15 μmol/L. HHcy is recognized as a potent risk factor and predictor for cardiovascular disease (CVD) in general populations^25^,^26^,^27^,^28^. Previously, a dose-response relationship between Hcy and CVD was identified in a population with plasma Hcy levels starting from 10 μmol/L^29^,^30^,^31^,^32^,^33^. In meta-analyses, the OR for cardiovascular disease was calculated to be 1.6 for a 5 μmol/L increase in plasma Hcy concentration^34^,^35^. The present study found that plasma Hcy levels, in a range from 5 to 20 μmol/L, were associated with a rapid decline of renal function and incident CKD in a hypertensive population. Increased risk of composite renal outcomes was found across Hcy tertiles, with a significantly increased OR at Hcy levels of approximately 13 μmol/L (in women) and 16 μmol/L (in men). This data is consistent with previous studies showing an increased risk of incident CKD and microalbuminuria in the general population with circulating Hcy levels of approximately 8 ~ 10 μmol/L^7^,^8^. These results indicate that an elevated plasma Hcy level, even in the range below the cutoff for HHcy, is a risk factor for renal function and incident CKD in a hypertensive population.

Our study findings suggest that elevated Hcy levels might be a modifiable risk factor for CKD, and that preventive interventions should be considered for hypertensive adults with increased plasma Hcy levels. A number of strategies, including dietary modification of food intake components and supplementation with folic acid and vitamins B6 and B12, have been reported to be effective at lowering Hcy levels and reducing cardiovascular risk^36^. Folic acid and B vitamin supplementation may be considered as a simple and safe intervention to prevent renal function decline in hypertensive adults. Additional study including clinical trials is warranted to determine whether Hcy-lowering therapy can reduce the risk of renal function decline and incident CKD in hypertensive adults.

It is to our surprise that plasma folate and vitamin B12 levels did not predict or correlate with the incident CKD and rapid decline of eGFR (Fig. 1). These findings suggest that HHcy in a hypertensive population is not directly caused by a folate and/or vitamin B12 deficiency. It is possible that hypertension may directly cause renal damage, which then leads to impaired Hcy excretion and elevated plasma Hcy, an inverse causality. On the other hand, folate and B12 insufficiency may lead to enzymatic impairment in Hcy metabolic pathways, including remethylation, transsulfuration and adenosylation that may be involved in hypertensive HHcy^13^,^16^. It is well established that MTHFR and cystathionine beta-synthase (CBS) are key enzymes for Hcy remethylation and transsulfuration, and the major variants for human HHcy. Folic acid and B vitamin supplementation has been used as an effective strategy to lower plasma Hcy levels and reduce CVD risk in MTHFR and CBS variants^37^,^38^,^39^,^40^. Therefore, folic acid and B vitamin supplementation might still prove to be a useful therapeutic option to reduce CKD risk in hypertensive patients with HHcy.

Our study has some limitations. As it was conducted in Chinese hypertensive adults, caution is needed when generalizing our findings to other populations. This study did not have the data on microalbuminuria, an important marker for the development and progression of CKD, which limits our ability to estimate the true incidence of CKD in this study.

In conclusion, our study demonstrated that elevated plasma Hcy level is an independent predictor of rapid decline in renal function and incident CKD in a Chinese hypertensive population. Our findings, if further confirmed, may have important clinical and public health implications.

## Methods

### Study population

This community-based cohort study was approved by the Ethics Committees of the Institute of Biomedicine, Anhui Medical University. Investigation was performed in accordance with relevant guidelines and regulations. All participants were provided with written consent. The study was conducted in rural communities in Lianyungang, China. Eligible participants were those: (1) aged 45 to 75 years and (2) with hypertension (BP ≥ 140/90 mmHg or currently on antihypertensive therapy). Participants were excluded from the study if they had (1) confirmed CKD (i.e., glomerular filtration rate [GFR] <60 ml/min/per 1.73 m^2^) at time of screening; (2) diagnosed secondary hypertension; (3) a history of stroke, myocardial infarction, heart failure, coronary revascularization, and/or congenital or acquired heart diseases; (4) cancer; and/or (5) liver disease and/or severe mental disorders.

A total of 3,522 hypertensive adults were screened in 2008, and 2,518 (71.5%) were assessed at follow-up and participated in a subsequent scheduled visit after five years. Among the 2,518 participants, 131 were excluded from the analysis because of missing follow-up data. The final analysis included 2,387 participants.

### Data collection

Data collection at baseline and at the follow-up visit were conducted by trained research staff according to a standard operating procedure.

#### Blood pressure measurement

Resting blood pressures were measured using a mercury sphygmomanometer in a seated position after ≥10 minutes of rest. The mean of the three readings on the first baseline screening day was recorded and confirmed by three measurements on the second baseline screening day with an interval of at least one day. The mean of the three BP measurements on the second baseline screening day was used as the baseline BP for analyses.

#### Questionnaires and medical history

Questionnaires were administered to collect information on socio-demographic status, medical history and medications. Diabetes was defined as physician-diagnosed diabetes, or currently receiving hypoglycemic therapy, or fasting glucose level higher than 7.0 mmol/L.

#### Anthropometry

Anthropometric measurements, including height and weight, were taken using a standard operating procedure.

#### Blood Sample

Venous blood was drawn after overnight fasting during the baseline visit and the follow-up visit. Both plasma and serum samples were collected and stored at −80 °C. The samples were shipped by commercial cold chain transportation and measured in a central laboratory.

### Laboratory measurements

#### Measurement of plasma Hcy, folate and vitamin B12

Plasma Hcy, folate and vitamin B12 were measured by enzyme-cycling method using a commercial kit.

#### Measurement of estimated glomerular filtration

Serum creatinine was measured by enzymatic method (sarcosine oxidase-PAP) using a commercial kit (Beckman Coulter, Brea, CA, USA) according to the manufacturer’s protocol. The Beckman assay was calibrated to the Roche/Hitachi P module Creatinase Plus enzymatic assay (Roche Diagnostics, Basel, Switzerland), traceable to an isotope-dilution mass spectrometry assay at the National Institute of Standards and Technology^41^,^42^. eGFR was calculated using the two-level CKD-Epidemiology Collaboration (CKD-EPI) formula^43^.

#### Measurement of other parameters

Fasting glucose, total cholesterol, triglycerides and HDL-C were measured using a Hitachi 7020 Automatic Analyzer (Beckman Coulter, Brea, CA, USA).

### Renal outcome definitions

#### Incident CKD

was defined as follow-up eGFR <60 ml/min/per 1.73 m^2^. To avoid excluding participants with minor fluctuations in eGFR that may be due to “noise”, the definition also included a >1 ml/min/per 1.73 m^2^/year decrease in eGFR^44^.

#### Rate of eGFR decline

was calculated as the difference between the follow-up eGFR and baseline eGFR, which was divided by follow-up duration and expressed as ml/min/per 1.73 m^2^/year. A negative eGFR change indicates a fall in eGFR.

### Statistical analysis

All of the analyses were conducted using the statistical package R software, version 3.0.1^45^. Mean and standard deviation or median (25th, 75th percentile) and percentages were calculated for the description of population characteristics by gender. The differences in population characteristics between men and women were compared using the Student’s *t* tests, signed rank tests, or chi-square test as appropriate. Multivariable logistic regression was used to assess the relations of Hcy, folate and vitamin B12 with incident CKD. Multivariate linear regression models were used to evaluate the associations between Hcy, folate and vitamin B12 with change in kidney function. In these analyses, Hcy, folate and vitamin B12 were modeled both as continuous variables and as categorical variables (categorized into tertiles). Hcy was natural log-transformed to normalize its skewed distributions. Because of gender differences in Hcy and folate distribution, gender-specific tertiles of Hcy and folate were used. The multivariate models were adjusted for age, gender, diabetes, SBP, BMI, smoking, cholesterol, HDL-C, triglycerides, eGFR, and previous use of ACEIs/ARBs. Effect modification was tested using a multivariate regression by stratifying the potential risk factors for renal outcomes. Adjusted splines of generalized additive models were created to evaluate the associations between baseline Hcy, folate and vitamin B12 and the outcomes. All tests were two-tailed and P < 0.05 was considered significant.

## Additional Information

**How to cite this article**: Xie, D. *et al.* Hyperhomocysteinemia predicts renal function decline: a prospective study in hypertensive adults. *Sci. Rep.* **5**, 16268; doi: 10.1038/srep16268 (2015).

## Figures and Tables

**Table 1:** Baseline characteristics of study participants.

Characteristic	Total (n = 2387)	Men (n = 578)	Women (n = 1809)	P value
Age (year)	60 ± 7	61 ± 7	60 ± 7	<0.001
Body mass index (kg/m^2^)	25.7 ± 3.4	25.0 ± 3.3	26.0 ± 3.4	<0.001
Smoking (no.[%])^a^				<0.001
Never or occasional	1859 (78.3)	167 (29.3)	1692 (93.8)	
Former	131 (5.5)	97 (17.0)	34 (1.9)	
Current	383 (16.1)	306 (53.7)	77 (4.3)	
Diabetes (no.[%])^b^	335 (14.0)	80 (13.8)	255 (14.1)	0.932
Systolic blood pressure (mmHg)	168 ± 20	167 ± 21	169 ± 20	0.070
Diastolic blood pressure (mmHg)	95 ± 11	97 ± 13	94 ± 11	<0.001
Laboratory variables				
Cholesterol (mmol/L)	5.84 ± 1.18	5.65 ± 1.16	5.90 ±1.18	<0.001
HDL-C (mmol/L)	1.36 ± 0.37	1.38 ± 0.42	1.36 ± 0.36	0.223
Triglycerides (mmol/L)	1.73 ± 0.94	1.60 ± 1.05	1.77 ± 0.90	<0.001
Hcy (μmol/L) (median [Q1-Q3])	12.1 (10.0,15.1)	14.3 (12.0,18.1)	11.5 (9.6, 14.0)	<0.001
Folate (nmol/L) (median [Q1-Q3])	7.4 (5.3,9.6)	5.9 (4.7,8.2)	7.9 (5.6,9.9)	<0.001
B12 (pg/ml) (median [Q1-Q3])	382.1 (322.1, 470.4)	366.4 (313.1,452.9)	385.5 (325.1, 476.7)	0.270
No. of antihypertensive drugs (median [Q1-Q3])	0 (0-1)	0 (0-1)	0 (0-1)	0.018
Use of ACEIs/ARBs (no.[%])	137 (5.7)	45 (7.8)	92 (5.1)	0.020
Use of Calcium channel blocker (no.[%])	140 (5.9)	39 (6.8)	101 (5.6)	0.331
Use of diuretic (no.[%])	69 (2.9)	11 (1.9)	58 (3.2)	0.143
Use of blocker (no.[%])	31 (1.3)	3 (0.5)	28 (1.6)	0.093
Baseline eGFR (ml/min/1.73 m^2^)^c^	93.9 ± 11.7	92.5 ± 11.6	94.4 ± 11.7	<0.001
eGFR at follow-up (ml/min/1.73 m^2^)^c^	89.6 ± 13.1	88.4 ± 13.2	90.0 ± 13.1	0.011
eGFR change (ml/min/1.73 m^2^/year)	−0.98 ± 2.33	−0.93 ± 2.3	−1.00 ± 2.4	0.524
Incident CKD at follow-up (no.[%])^d^	69 (2.9)	19 (3.3)	50 (2.8)	0.609

Data is presented as mean (median) and standard deviation (interquartile range) for continuous variables and percentage for dichotomous variables, respectively.

Abbreviations: ACEIs, angiotensin converting enzyme inhibitors; ARBs, angiotensin II receptor blockers; CKD, chronic kidney disease; eGFR, estimated glomerular filtration rate; Hcy, homocysteine; HDL-C, high density lipoprotein cholesterol.

^a^Smoking status was classed as never, occasional (<10 packs total), former (≥10 packs total but none in the past year) and current.

^b^Diabetes was defined as physician-diagnosed diabetes, or currently receiving hypoglycemic therapy, or fasting glucose level higher than 7.0 mmol/L.

^c^eGFR was estimated using the CKD-Epidemiology Collaboration equation.

^d^Incident CKD was defined as follow-up eGFR <60 ml/min/1.73 m^2^ and eGFR decline rate >1 ml/min/1.73 m^2^/year.

**Table 2:** Association of baseline homocysteine, folate and vitamin B12 with incident chronic kidney disease^a^.

Variables	Subjects (N)	CKD Events (n[%])	Crude Model		Adjusted Model^b^		Hcy, FA, vitamin B12 Fully Adjusted Model^c^	
			OR (95% CI)	P	OR (95% CI)	P	OR (95% CI)	P
Homocysteine (μmol/L)								
Continuous (log transformed)	2387	69 (2.9)	3.32 (2.12 ~ 5.21)	<0.001	2.17 (1.15 ~ 4.10)	0.017	2.44 (1.26 ~ 4.72)	0.008
Tertiles:								
Tertile 1 (men<12.8 μM/L, women <10.2 μMmol/L)	796	7 (0.9)	1 (ref)	–	1 (ref)	–	1 (ref)	–
Tertile 2 (men12.8–16.5 μM/L, women 10.2–13.1 μMmol/L)	793	16 (2.0)	2.32 (0.95 ~ 5.67)	0.065	1.29 (0.51 ~ 3.29)	0.591	1.50 (0.55 ~ 4.1)	0.428
Tertile 3 (men>16.5 μM/L, women >13.1 μM/L)	798	46 (5.8)	6.89 (3.09 ~ 15.37)	<0.001	2.46 (1.05 ~ 5.77)	0.038	3.23 (1.25 ~ 8.35)	0.016
*P for trend*				<0.001		0.011		0.003
Folate (nmol/L)								
Continuous (per 5 nmol/L increase)	2296	68 (3.0)	0.72 (0.47 ~ 1.10)	0.129	0.83 (0.54 ~ 1.28)	0.409	0.92 (0.62 ~ 1.37)	0.685
Tertiles:								
Tertile 1 (men<5.1 nM/L, women<6.3 nM/L)	765	26 (3.4)	1 (ref)	−	1 (ref)	−	1 (ref)	–
Tertile 2 (men5.1 ~ 7.2 nM/L, women:6.3 ~ 9.2 nM/L)	765	23 (3.0)	0.88 (0.50 ~ 1.56)	0.663	1.06 (0.57 ~ 1.97)	0.849	1.17 (0.63 ~ 2.20)	0.618
Tertile 3 (men>7.2 nM/L, women >9.2 nM/L)	766	19 (2.5)	0.72 (0.40~1.32)	0.289	0.99 (0.52 ~ 1.90)	0.974	1.31 (0.67 ~ 2.59)	0.431
*P for trend*				0.290		0.985		0.422
Vitamin B12 (pg/ml)								
Continuous (per 100 pg/ml increase)	2296	68 (3.0)	1.04 (0.91 ~ 1.20)	0.542	1.03 (0.89 ~ 1.19)	0.681	1.08 (0.94 ~ 1.24)	0.283
Tertiles:								
Tertile 1 (<341.7 pg/ml)	765	20 (2.6)	1 (ref)	–	1 (ref)	–	1 (ref)	–
Tertile 2 (341.7 ~ 430.2pg/ml)	765	29 (3.8)	1.47 (0.82 ~ 2.62)	0.194	1.41 (0.75 ~ 2.66)	0.283	1.63 (0.86 ~ 3.09)	0.138
Tertile 3 (>430.2 pg/ml)	766	19 (2.5)	0.95 (0.50 ~ 1.79)	0.868	0.88 (0.44 ~ 1.77)	0.728	1.17 (0.56 ~ 2.41)	0.678
*P for trend*				0.877		0.708		0.655

Abbreviations: ACEIs, angiotensin converting enzyme inhibitors; ARBs, angiotensin II receptor blockers; CI, confidence interval; eGFR, estimated glomerular filtration rate; FA, folate; Hcy, homocysteine; HDL-C, high density lipoprotein cholesterol; SBP, systolic blood pressure.

^a^Incident chronic kidney disease was defined as follow-up eGFR <60 ml/min/1.73 m^2^ and eGFR decline rate >1 ml/min/1.73 m^2^/year.

^b^Homocysteine, folate and vitamin B12 were individually included in the multivariate model as continuous or categorical variables. Model was adjusted for age, gender, SBP, diabetes, smoking, cholesterol, HDL-C, triglycerides, body mass index, eGFR, previous use of ACEIs/ARBs, and SBP at the endpoint of the follow-up.

^c^Homocysteine, folate and vitamin B12 were all included in the multivariate model as continuous or categorical variables. Model was adjusted for age, gender, SBP, diabetes, smoking, cholesterol, HDL-C, triglycerides, body mass index, eGFR, previous use of ACEIs/ARBs, and SBP at the endpoint of the follow-up.

**Table 3:** Association of baseline homocysteine, folate and vitamin B12 with eGFR decline rate during follow-up^a^.

Variable	Subjects (N)	eGFR decline rate (ml/min/1.73 m^2^/year) (median [Q1, Q3])	Crude Model		Adjusted Model^b^		Hcy, FA, vitamin B12 Fully Adjusted Model^c^	
			β (SE)	P	β (SE)	P	β (SE)	P
Homocysteine (μmol/L)								
Continuous (log transformed)	2387	−0.87 (−2.04, 0.20)	0.12 (0.13)	0.347	−0.42 (0.13)	0.001	−0.46 (0.14)	<0.001
Tertiles:								
Tertile 1 (men<12.8 μM/L, women <10.2 μMmol/L)	796	−0.87 (−1.92, 0.06)	0 (ref)	−	0 (ref)	–	0 (ref)	–
Tertile 2 (men12.8–16.5 μM/L, women 10.2–13.1 μMmol/L)	793	−0.89 (−1.93, 0.07)	0.02 (0.12)	0.868	−0.17 (0.11)	0.117	−0.16 (0.11)	0.160
Tertile 3 (men>16.5 μM/L, women >13.1 μM/L)	798	−0.84 (−2.23, 0.48)	0.04 (0.12)	0.729	−0.45 (0.11)	<0.001	−0.48 (0.12)	<0.001
* P for trend*				0.729		<0.001		<0.001
Folate (nmol/L)								
Continuous (per 5 nmol/L increase)	2296	−0.87 (−2.01, 0.21)	0.06 (0.07)	0.402	0.07 (0.07)	0.332	0.02 (0.07)	0.785
Tertiles:								
Tertile 1 (men<5.1 nM/L, women<6.3 nM/L)	765	−0.92 (−1.98, 0.09)	0 (ref)	–	0 (ref)	–	0 (ref)	–
Tertile 2 (men5.1~7.2 nM/L, women: 6.3 ~ 9.2 nM/L)	765	−0.85(−1.96, 0.21)	0.12 (0.12)	0.311	0.08 (0.11)	0.461	0.02 (0.11)	0.869
Tertile 3 (men>7.2 nM/L, women >9.2 nM/L)	766	−0.84 (−2.08, 0.29)	0.07 (0.12)	0.556	0.05 (0.11)	0.628	−0.06 (0.11)	0.609
*P for trend*				0.556		0.629		0.640
Vitamin B12 (pg/ml)								
Continuous(per 100 pg/ml increase)	2296	−0.87 (−2.01, 0.21)	0.05 (0.03)	0.109	0.01 (0.03)	0.737	−0.01 (0.03)	0.634
Tertiles:								
Tertile 1 (<341.7 pg/ml)	765	−1.06 (−2.09, −0.12)	0 (ref)	–	0 (ref)	–	0 (ref)	–
Tertile 2 (341.7~430.2 pg/ml)	765	−0.80 (−2.13, 0.31)	0.26 (0.12)	0.030	0.12 (0.11)	0.285	0.05 (0.11)	0.676
Tertile 3 (>430.2 pg/ml)	766	−0.68 (−1.77, 0.46)	0.41 (0.12)	<0.001	0.21 (0.11)	0.057	0.08 (0.12)	0.475
*P for trend*				<0.001		0.057		0.476

Abbreviations: ACEIs, angiotensin converting enzyme inhibitors; ARBs, angiotensin II receptor blockers; CI, confidence interval; eGFR, estimated glomerular filtration rate; FA, folate; Hcy, homocysteine; HDL-C, high density lipoprotein cholesterol; SBP, systolic blood pressure.

^a^eGFR decline rate was calculated as the difference between the follow-up eGFR and baseline eGFR, which was divided by follow-up duration and expressed as ml/min/per 1.73 m^2^/year. Negative eGFR change indicates a fall in eGFR during follow-up.

^b^Homocysteine, folate and vitamin B12 were individually included in the multivariate model as continuous or categorical variables. Model was adjusted for age, gender, SBP, diabetes, smoking, cholesterol, HDL-C, triglycerides, body mass index, eGFR, previous use of ACEIs/ARBs, and SBP at the end of the follow-up.

^c^Homocysteine, folate and vitamin B12 were all included in the multivariate model as continuous or categorical variables. Model was adjusted for age, gender, SBP, diabetes, smoking, cholesterol, HDL-C, triglycerides, body mass index, eGFR, previous use of ACEIs/ARBs, and SBP at the end of the follow-up.

**Table 4:** Stratified analysis for association of homocysteine, folate and vitamin B12 with renal outcome.

Variable	Homocysteine (per log1 μmol/L)		Folate (per 5 nmol/L)		B12 (per 100 pg/ml)	
	OR (95% CI) or β (SE)^c^	P	OR (95% CI) or β (SE)^c^	P	OR (95% CI) or β (SE)^c^	P
Incident CKD^a^						
Men	2.02 (0.56 ~ 7.34)	0.285	0.69 (0.20 ~ 2.39)	0.555	1.12 (0.80 ~ 1.58)	0.504
Women	2.95 (1.30,6.73)	0.010	0.99 (0.65 ~ 1.52)	0.976	1.12 (0.95 ~ 1.32)	0.179
Age<60 years	3.26 (0.74 ~ 14.34)	0.118	0.69 (0.20 ~ 2.43)	0.567	1.34 (1.03 ~ 1.74)	0.030
Age≥60 years	2.21 (1.04 ~ 4.68)	0.039	1.00 (0.65 ~ 1.55)	0.998	1.02 (0.85 ~ 1.23)	0.830
Baseline SBP<160 mmHg	3.49 (0.81 ~ 15.09)	0.094	0.55 (0.23 ~ 1.32)	0.181	1.33 (1.07 ~ 1.65)	0.011
Baseline SBP≥160 mmHg	1.73 (0.77 ~ 3.88)	0.184	0.89 (0.54 ~ 1.48)	0.664	0.82 (0.61 ~ 1.12)	0.211
eGFR>90 ml/min/1.73 m^2^	0.66 (0.07 ~ 6.63)	0.726	1.21 (0.72 ~ 2.05)	0.466	1.11 (0.83 ~ 1.48)	0.482
eGFR 60 ~ 90 ml/min/1.73 m^2^	2.77 (1.33 ~ 5.77)	0.006	0.81 (0.50 ~ 1.31)	0.392	1.07 (0.89 ~ 1.28)	0.477
eGFR decline^b^						
Men	−0.35 (0.22)	0.116	−0.15 (0.17)	0.358	0.04 (0.06)	0.440
Women	−0.57 (0.18)	0.001	0.05 (0.08)	0.499	−0.04 (0.04)	0.304
Age<60 years	−0.52 (0.19)	0.005	0.07 (0.11)	0.494	−0.08 (0.05)	0.101
Age≥60 years	−0.39 (0.20)	0.056	−0.05 (0.09)	0.631	0.03 (0.04)	0.438
Baseline SBP<160 mmHg	−0.40 (0.25)	0.103	0.03 (0.12)	0.835	−0.06 (0.05)	0.184
Baseline SBP≥160 mmHg	−0.47 (0.17)	0.005	0.02 (0.09)	0.782	0.02 (0.04)	0.555
eGFR>90 ml/min/1.73 m^2^	−0.29 (0.15)	0.048	−0.05 (0.07)	0.529	0.01 (0.03)	0.864
eGFR 60 ~ 90 ml/min/1.73 m^2^	−0.81 (0.29)	0.005	0.17 (0.15)	0.277	−0.05 (0.06)	0.358

Abbreviations: ACEIs, angiotensin converting enzyme inhibitors; ARBs, angiotensin II receptor blockers; CI, confidence interval; eGFR, estimated glomerular filtration rate; FA, folate; Hcy, homocysteine; HDL-C, high density lipoprotein cholesterol; SBP, systolic blood pressure.

^a^Incident chronic kidney disease was defined as follow-up eGFR <60 ml/min/1.73 m^2^ and eGFR decline rate >1 ml/min/1.73 m^2^/year.

^b^eGFR decline rate was calculated as the difference between the follow-up eGFR and baseline eGFR, which was divided by follow-up duration and expressed as ml/min/per 1.73 m^2^/year. Negative eGFR change indicates a fall in eGFR during follow-up.

^c^Homocysteine, folate and vitamin B12 were all included in the multivariate model as continuous variables. Model was adjusted for age, gender, SBP, diabetes, smoking, cholesterol, HDL-C, triglycerides, body mass index, eGFR, previous use of ACEIs/ARBs, and SBP at the end of the follow-up.

## References

[b1] Coresh J. *et al.* Prevalence of chronic kidney disease in the United States. JAMA 298, 2038–2047 (2007). 17986697 10.1001/jama.298.17.2038

[b2] Zhang Q. L. & Rothenbacher D. Prevalence of chronic kidney disease in population-based studies: systematic review. BMC Public Health 8, 117 (2008). 18405348 10.1186/1471-2458-8-117 PMC2377260

[b3] Zhang L. *et al.* Prevalence of chronic kidney disease in China: a cross-sectional survey. Lancet 379, 815–822 (2012). 22386035 10.1016/S0140-6736(12)60033-6

[b4] Hsu C. Y., McCulloch C. E., Darbinian J., Go A. S. & Iribarren C. Elevated blood pressure and risk of end-stage renal disease in subjects without baseline kidney disease. Arch Intern Med 165, 923–928 (2005). 15851645 10.1001/archinte.165.8.923

[b5] Tozawa M. *et al.* Blood pressure predicts risk of developing end-stage renal disease in men and women. Hypertension 41, 1341–1345 (2003). 12707291 10.1161/01.HYP.0000069699.92349.8C

[b6] Klag M. J. *et al.* Blood pressure and end-stage renal disease in men. N Engl J Med 334, 13–18 (1996). 7494564 10.1056/NEJM199601043340103

[b7] Ninomiya T. *et al.* Hyperhomocysteinemia and the development of chronic kidney disease in a general population: the Hisayama study. Am J Kidney Dis 44, 437–445 (2004). 15332216

[b8] Fox C. S. *et al.* A multi-marker approach to predict incident CKD and microalbuminuria. J Am Soc Nephrol 21, 2143–2149 (2010). 20966127 10.1681/ASN.2010010085 PMC3014027

[b9] Shastry S., Ingram A. J., Scholey J. W. & James L. R. Homocysteine induces mesangial cell apoptosis via activation of p38-mitogen-activated protein kinase. Kidney Int 71, 304–311 (2007). 17149372 10.1038/sj.ki.5002031

[b10] Yi F. *et al.* Inhibition of ceramide-redox signaling pathway blocks glomerular injury in hyperhomocysteinemic rats. Kidney Int 70, 88–96 (2006). 16688115 10.1038/sj.ki.5001517

[b11] Sethi A. S., Lees D. M., Douthwaite J. A., Dawnay A. B. & Corder R. Homocysteine-induced endothelin-1 release is dependent on hyperglycaemia and reactive oxygen species production in bovine aortic endothelial cells. J Vasc Res 43, 175–183 (2006). 16410680 10.1159/000090947

[b12] Yi F. & Li P. L. Mechanisms of homocysteine-induced glomerular injury and sclerosis. Am J Nephrol 28, 254–264 (2008). 17989498 10.1159/000110876 PMC2820346

[b13] Lee M. E. & Wang H. Homocysteine and hypomethylation. A novel link to vascular disease. Trends Cardiovasc Med 9, 49–54 (1999). 10189967 10.1016/s1050-1738(99)00002-x

[b14] Marti F. *et al.* Hyperhomocysteinemia is independently associated with albuminuria in the population-based CoLaus study. BMC Public Health 11, 733 (2011). 21943240 10.1186/1471-2458-11-733 PMC3188498

[b15] Jager A. *et al.* Serum homocysteine levels are associated with the development of (micro)albuminuria: the Hoorn study. Arterioscler Thromb Vasc Biol 21, 74–81 (2001). 11145936 10.1161/01.atv.21.1.74

[b16] Chen N. C. *et al.* Regulation of homocysteine metabolism and methylation in human and mouse tissues. FASEB J 24, 2804–2817 (2010). 20305127 10.1096/fj.09-143651 PMC2909276

[b17] Frosst P. *et al.* A candidate genetic risk factor for vascular disease: a common mutation in methylenetetrahydrofolate reductase. Nat Genet 10, 111–113 (1995). 7647779 10.1038/ng0595-111

[b18] Stampfer M. J. & Malinow M. R. Can lowering homocysteine levels reduce cardiovascular risk? N Engl J Med 332, 328–329 (1995). 7654269 10.1056/NEJM199502023320511

[b19] Rimm E. B. *et al.* Folate and vitamin B6 from diet and supplements in relation to risk of coronary heart disease among women. JAMA 279, 359–364 (1998). 9459468 10.1001/jama.279.5.359

[b20] Gao Y. *et al.* Prevalence of hypertension in china: a cross-sectional study. PLoS One 8, e65938 (2013). 23776574 10.1371/journal.pone.0065938 PMC3679057

[b21] Hao L. *et al.* High prevalence of hyperhomocysteinemia in Chinese adults is associated with low folate, vitamin B-12, and vitamin B-6 status. J Nutr 137, 407–413 (2007). 17237319 10.1093/jn/137.2.407

[b22] Hao L. *et al.* Geographical, seasonal and gender differences in folate status among Chinese adults. J Nutr 133, 3630–3635 (2003). 14608086 10.1093/jn/133.11.3630

[b23] Botto L. D. & Yang Q. 5,10-Methylenetetrahydrofolate reductase gene variants and congenital anomalies: a HuGE review. Am J Epidemiol 151, 862–877 (2000). 10791559 10.1093/oxfordjournals.aje.a010290

[b24] Huo Y. *et al.* Efficacy of folic acid therapy in primary prevention of stroke among adults with hypertension in China: the CSPPT randomized clinical trial. JAMA 313, 1325–1335 (2015). 25771069 10.1001/jama.2015.2274

[b25] Veeranna V. *et al.* Homocysteine and reclassification of cardiovascular disease risk. J Am Coll Cardiol 58, 1025–1033 (2011). 21867837 10.1016/j.jacc.2011.05.028

[b26] Kang S. S., Wong P. W. & Malinow M. R. Hyperhomocyst(e)inemia as a risk factor for occlusive vascular disease. Annu Rev Nutr 12, 279–298 (1992). 1503807 10.1146/annurev.nu.12.070192.001431

[b27] Stanger O. *et al.* Clinical use and rational management of homocysteine, folic acid, and B vitamins in cardiovascular and thrombotic diseases. Z Kardiol 93, 439–453 (2004). 15252738 10.1007/s00392-004-0075-3

[b28] Towfighi A., Markovic D. & Ovbiagele B. Pronounced association of elevated serum homocysteine with stroke in subgroups of individuals: a nationwide study. J Neurol Sci 298, 153–157 (2010). 20810133 10.1016/j.jns.2010.07.013

[b29] Bostom A. G. *et al.* Nonfasting plasma total homocysteine levels and stroke incidence in elderly persons: the Framingham Study. Ann Intern Med 131, 352–355 (1999). 10475888 10.7326/0003-4819-131-5-199909070-00006

[b30] Boushey C. J., Beresford S. A., Omenn G. S. & Motulsky A. G. A quantitative assessment of plasma homocysteine as a risk factor for vascular disease. Probable benefits of increasing folic acid intakes. JAMA 274, 1049–1057 (1995). 7563456 10.1001/jama.1995.03530130055028

[b31] Malinow M. R., Nieto F. J., Szklo M., Chambless L. E. & Bond G. Carotid artery intimal-medial wall thickening and plasma homocyst(e)ine in asymptomatic adults. The Atherosclerosis Risk in Communities Study. Circulation 87, 1107–1113 (1993). 8462139 10.1161/01.cir.87.4.1107

[b32] Nygard O. *et al.* Plasma homocysteine levels and mortality in patients with coronary artery disease. N Engl J Med 337, 230–236 (1997). 9227928 10.1056/NEJM199707243370403

[b33] Wilcken D. E. & Wilcken B. The natural history of vascular disease in homocystinuria and the effects of treatment. J Inherit Metab Dis 20, 295–300 (1997). 9211201 10.1023/a:1005373209964

[b34] den Heijer M., Rosendaal F. R., Blom H. J., Gerrits W. B. & Bos G. M. Hyperhomocysteinemia and venous thrombosis: a meta-analysis. Thromb Haemost 80, 874–877 (1998). 9869152

[b35] Wald D. S., Law M. & Morris J. K. Homocysteine and cardiovascular disease: evidence on causality from a meta-analysis. BMJ 325, 1202 (2002). 12446535 10.1136/bmj.325.7374.1202 PMC135491

[b36] Wang X. *et al.* Efficacy of folic acid supplementation in stroke prevention: a meta-analysis. Lancet 369, 1876–1882 (2007). 17544768 10.1016/S0140-6736(07)60854-X

[b37] Jiang X. *et al.* Hyperhomocystinemia impairs endothelial function and eNOS activity via PKC activation. Arterioscler Thromb Vasc Biol 25, 2515–2521 (2005). 16210565 10.1161/01.ATV.0000189559.87328.e4 PMC4400833

[b38] Jamaluddin M. D. *et al.* Homocysteine inhibits endothelial cell growth via DNA hypomethylation of the cyclin A gene. Blood 110, 3648–3655 (2007). 17698632 10.1182/blood-2007-06-096701 PMC2077313

[b39] Cheng Z., Yang X. & Wang H. Hyperhomocysteinemia and Endothelial Dysfunction. Curr Hypertens Rev 5, 158–165 (2009). 20495681 10.2174/157340209788166940 PMC2873778

[b40] Qin X. *et al.* Homocysteine-lowering therapy with folic acid is effective in cardiovascular disease prevention in patients with kidney disease: a meta-analysis of randomized controlled trials. Clin Nutr 32, 722–727 (2013). 23313356 10.1016/j.clnu.2012.12.009

[b41] Levey A. S. *et al.* Expressing the modification of diet in renal disease study equation for estimating glomerular filtration rate with standardized serum creatinine values. Clin Chem 53, 766–772 (2007). 17332152 10.1373/clinchem.2006.077180

[b42] Junge W., Wilke B., Halabi A. & Klein G. Determination of reference intervals for serum creatinine, creatinine excretion and creatinine clearance with an enzymatic and a modified Jaffe method. Clin Chim Acta 344, 137–148 (2004). 15149882 10.1016/j.cccn.2004.02.007

[b43] Levey A. S. *et al.* A new equation to estimate glomerular filtration rate. Ann Intern Med 150, 604–612 (2009). 19414839 10.7326/0003-4819-150-9-200905050-00006 PMC2763564

[b44] Madero M. *et al.* Association of arterial rigidity with incident kidney disease and kidney function decline: the Health ABC study. Clin J Am Soc Nephrol 8, 424–433 (2013). 23271800 10.2215/CJN.07900812 PMC3586973

[b45] Ford E. S. & Bowman B. A. Serum and red blood cell folate concentrations, race, and education: findings from the third National Health and Nutrition Examination Survey. Am J Clin Nutr 69, 476–481 (1999). 10075333 10.1093/ajcn/69.3.476

